# Advanced HIV disease and its predictors among newly diagnosed PLHIV in the Gedeo zone, southern Ethiopia

**DOI:** 10.1371/journal.pone.0310373

**Published:** 2024-09-13

**Authors:** Temesgen Leka Lerango, Tesfalidet Markos, Daniel Yehualeshet, Endashaw Kefyalew, Semalgn Leka Lerango

**Affiliations:** 1 School of Public Health, College of Medicine and Health Sciences, Dilla University, Dilla, Ethiopia; 2 Department of Midwifery, College of Medicine and Health Sciences, Dilla University, Dilla, Ethiopia; 3 School of Medicine, College of Medicine and Health Sciences, Dilla University, Dilla, Ethiopia; 4 School of Medicine, College of Medicine and Health Sciences, Addis Ababa University, Addis Ababa, Ethiopia; London School of Hygiene and Tropical Medicine, UNITED KINGDOM OF GREAT BRITAIN AND NORTHERN IRELAND

## Abstract

**Background:**

Globally, HIV infection remains a leading cause of morbidity and mortality. Despite reducing new infections, the global response to advanced HIV disease (AHD) remains ineffective, leaving HIV epidemics a significant public health threat worldwide. In Ethiopia, evidence regarding AHD is scarce. Therefore, this study aimed to assess the prevalence and predictors of AHD among newly diagnosed people living with HIV (PLHIV) initiating antiretroviral therapy in the Gedeo zone, southern Ethiopia.

**Methods:**

A facility-based cross-sectional study was conducted from May 29, 2023, to February 06, 2024, at health facilities providing HIV care in the Gedeo zone, southern Ethiopia. A total of 427 PLHIV-initiating antiretroviral therapy (ART) were recruited for the study. The data were collected through face-to-face interviews and record reviews using KoboCollect version 2.4 and analyzed using R version 4.3.3. The Akaike information criterion (AIC) model selection was used to evaluate and choose the best-fitting model to describe the relationship between AHD and predictors. Finally, variables with a p-value less than 0.05 were considered independent predictors in the multivariable regression analysis.

**Results:**

The study participants’ mean (±SD) age was 31.3 (±8.7) years. The overall prevalence of AHD among newly diagnosed PLHIV-initiating ART was 34.4% (95% CI: 29.8%, 39.1%). Rural residence (AOR = 3.48, 95% CI: 2.24, 5.47), alcohol consumption (AOR = 2.48, 95% CI: 1.59, 3.90), and being identified through community-based index case testing (ICT) (AOR = 0.26, 95% CI: 0.13, 0.51) were found to be independent predictors of AHD.

**Conclusions:**

The prevalence of AHD among newly diagnosed individuals initiating ART was high. PLHIV who consume alcohol should receive detailed counseling on how it can negatively impact their progress with antiretroviral treatment. HIV testing should be enhanced in rural communities by strengthening community health campaigns. Furthermore, community-based index case testing should be strengthened for early identification of PLHIV.

## Introduction

In 2023, 39.9 million people were living with HIV (PLHIV) worldwide. Of these, 3.3% (1.3 million) were newly infected with HIV. Moreover, around 630,000 people died from AIDS-related illnesses worldwide in 2023. This figure significantly exceeds the target of fewer than 250,000 deaths by 2025 [1]. Globally, and particularly in sub-Saharan Africa, HIV infection remains a leading cause of morbidity and mortality [2].

The proportion of PLHIV receiving treatment increased from 17 million at the end of 2015 [3] to 30.7 million (77% of all PLHIV) at the end of 2023. Since 2010, new HIV infections have decreased by 39%, from 2.1 million to 1.3 million in 2023 [1]. Even with this progress, about half of people living with HIV (PLHIV) still present to healthcare with advanced HIV disease (AHD) [4]. Antiretroviral treatment (ART) began in Ethiopia in 2003, and free ART was launched in 2005 [5]. ART services are provided in more than 1,500 public, private, and NGO health facilities [6]. In 2023, there were an estimated 610,000 PLHIV. The number of new infections was 7,400, and the number of AIDS-related deaths was 10,000. Among PLHIV, 85% (510,000 individuals) were receiving ART [7].

The international community is committed to ending the AIDS epidemic as a public health threat by 2030 [8]. To achieve this goal, the world is implementing a Fast-Track strategy. The aim was to ensure that by 2030, 95% of people living with HIV know their HIV status, 95% of those who know their status are receiving antiretroviral therapy, and 95% of those on treatment have a suppressed viral load. Meeting these Fast-Track targets will effectively end the AIDS epidemic [9]. As of 2023, these achievements were 86%, 89%, and 93% worldwide [1, 10], and 90%, 85%, and 75% in Ethiopia, respectively [7].

The U.S. President’s Emergency Plan for AIDS Relief (PEPFAR) and other stakeholders have significantly reduced incident HIV cases in low-income countries by emphasizing HIV prevention, case detection, and provision of antiretroviral therapy [11]. Despite the positive effect on reducing new infections, the global response to AHD remains ineffective, leaving HIV epidemics a significant public health threat in all regions of the world [3, 12]. It has been reported that 30–40% of PLHIV starting ART in low- and middle-income settings have Clusters of Differentiation 4 (CD4) cell count of less than 200 cells/mm^3^, while 20% have a CD4 cell count of less than 100 cells/mm^3^ [4].

The World Health Organization (WHO) defined AHD as having a CD4 cell count of <200 cells/mm^3^ or being at WHO clinical stage 3 or 4 disease for children five years and older, adolescents, and adults while all children under five years living with HIV are considered to have advanced HIV disease [13]. In the coming years, responding to AHD will remain an important public health priority [2]. People with AHD face a high risk of death, even after starting ART, and this risk increases as their CD4 cell count decreases [6, 14]. The risk of death has remained relatively constant in recent years due to the progression to AHD [15].

AHD is also associated with unwanted healthcare costs, increased viral reservoirs, increased risk of opportunistic infections (OIs), increased inflammation, immune reconstitution inflammatory syndrome, and increased risk of AIDS-related and non-AIDS-related comorbidities [13]. Older age, male sex, level of education, overweight body mass index, consideration for HIV testing, personal health perception barrier, seeking treatment from traditional healers, and sample sources were reported to be factors associated with AHD among PLHIV-initiating ART [16–21].

HIV prevention progress lags far behind what is needed. In low- and middle-income countries, the HIV response is hindered by a funding gap that is growing. The AIDS epidemic can be ended if leaders make the right political and financial choices [10]. Intensified effort is needed for the early identification of PLHIV followed by a linkage to ART care [22]. The intervention package for AHD including screening, early ART initiation, prevention and/or management for major OIs, and adherence support should be given to all individuals presenting with advanced HIV disease [4] using a differentiated service delivery approach [5]. The implementation of the WHO’s package of care interventions for AHD is feasible, but its performance remains low in many countries [23]. The world can end AIDS as a public health threat only by overcoming it everywhere and for everyone [10].

In Ethiopia, evidence is scarce regarding the burden and factors contributing to advanced HIV disease in newly diagnosed PLHIV who are initiating ART. Therefore, this study aimed to assess the prevalence and predictors of advanced HIV disease among newly diagnosed people living with HIV-initiating ART in the Gedeo zone, southern Ethiopia. The findings will assist in designing interventions to reduce the number of PLHIV who initiate ART at an advanced stage. They will also help caregivers and stakeholders identify key focus areas in HIV care targeted at addressing AHD.

## Materials and methods

### Study setting, design, and period

Gedeo is a zone in the South Ethiopia Regional State (SERS) of Ethiopia. It is surrounded by the Oromia Region, which borders the zone on the east, south, and west. Gedeo shares its northern boundary with the Sidama Region. Dilla, located on the main road from Addis Ababa to Nairobi, is the administrative center. The 2007 Census of Ethiopia reports that this zone has a total population of 847,434, of whom 424,742 are men and 422,692 are women. The four largest ethnic groups reported in this zone were the Gedeo (86.14%), the Oromo (4.71%), the Amhara (3.37%), and the Gurage (1.55%). The religious practices of the residents are Protestant (73.21%), Ethiopian Orthodox Christianity (10.67%), traditional religions (7.96%), Islam (2.44%), and Catholic (2.11%).

A cross-sectional study was conducted from May 29, 2023, to February 06, 2024, at health facilities providing HIV care in the Gedeo zone, southern Ethiopia. Dilla University Referral Hospital, Bule Primary Hospital, Gedeb Primary Hospital, Yirgachefe Primary Hospital, Dilla Health Center, Wonago Health Center, Gerse Health Center, and Chelelktu Health Center provide HIV care in the Gedeo zone.

### Study population

All newly diagnosed people living with HIV (PLHIV) aged 18 years or older, who were starting antiretroviral therapy (ART) in the Gedeo zone, were included. In contrast, PLHIV who were transferring in from other facilities and those who were representing or re-engaging in ART care were excluded from the study.

### Sample size determination

The sample size for this study was determined using a single population proportion formula, taking into account a 50% prevalence of advanced HIV disease among newly diagnosed PLHIV-initiating ART, a 95% confidence interval, a 5% degree of precision, and a z-value at a 95% confidence interval of 1.96. The computed result was 384. After accounting for a 10% nonresponse rate, the final determined sample size was 427.

### Participant recruitment

**Fig 1:**
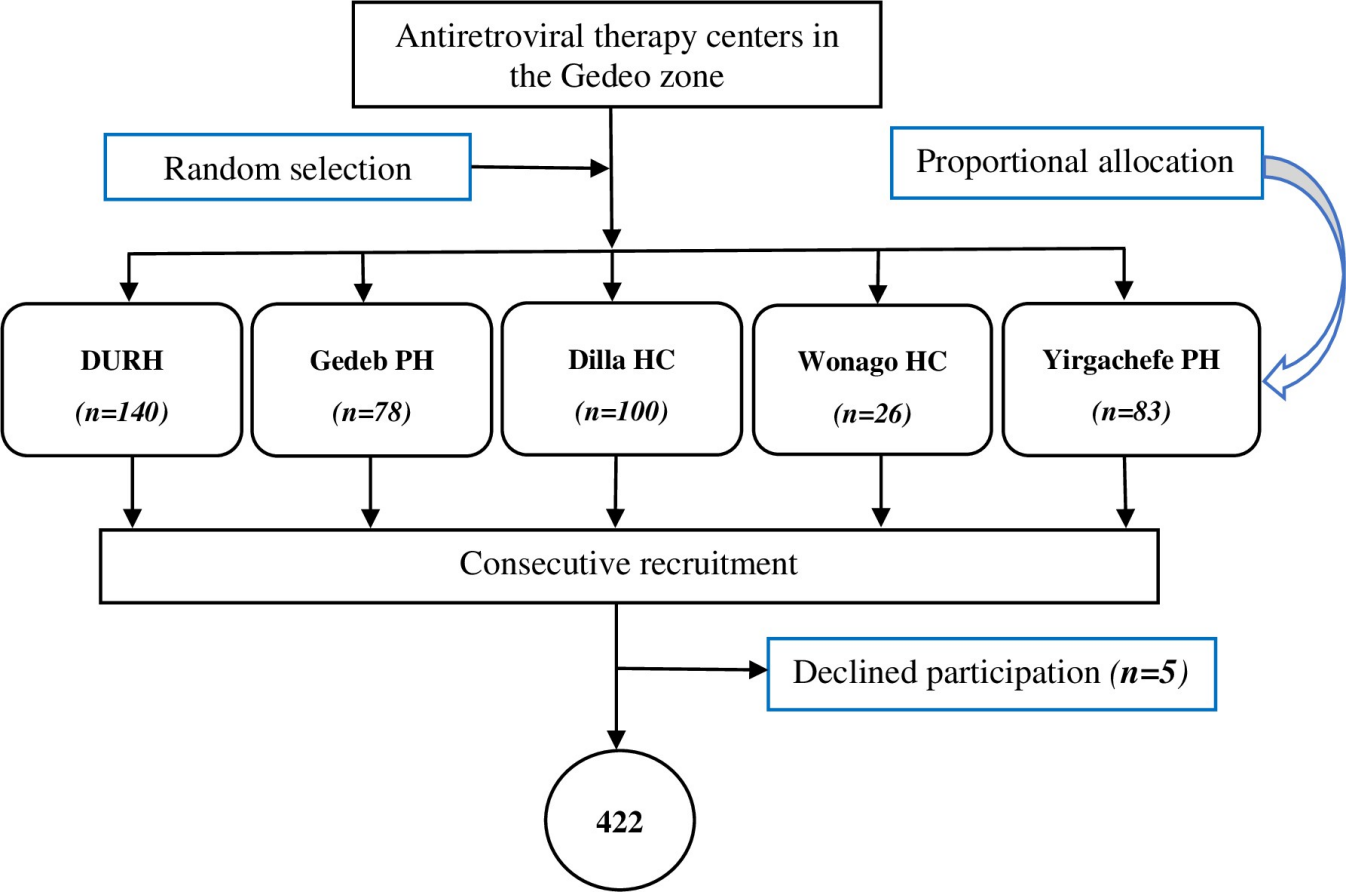
Schematic representation of the recruitment process.

Five of the eight facilities providing ART care in the Gedeo zone were selected randomly. According to previous records, the annual average number of PLHIV initiating ART at HIV care facilities in the Gedeo zone is as follows: Dilla University Referral Hospital (N = 181), Yirgachefe Primary Hospital (N = 93), Gedeb Primary Hospital (N = 89), Dilla Health Center (N = 97), and Wonago Health Center (N = 32). Based on these data, participant recruitment was proportionally allocated to each facility. To ensure comprehensive data collection, including interview data and data abstraction from records, we recruited all newly diagnosed PLHIV who returned for a second engagement. Recruitment was conducted at health facilities and was independent of the test approach used. The method of initial HIV diagnosis for the patients could have been through routine testing at a health facility or as part of index case testing. HIV index case testing is a test offered to all index cases to elicit and test sexual partners and biological children of PLHIV. After getting consent from index cases, and integrating with health extension workers, health facilities conduct targeted community outreach to find and link elicited contacts of index cases (Fig 1).

### Variables of the study

#### Dependent variable

Advanced HIV disease was an outcome variable for this study. AHD is defined as a CD4 cell count of <200 cells/mm^3^ or being at WHO clinical stage 3 or 4.

#### Independent variables

Sociodemographic characteristics, clinical and health system-related characteristics, and psychosocial and behavioral characteristics constitute independent variables for the current study.

### Operational definition

#### Rapid ART initiation

ART initiation within seven days from the day of HIV diagnosis; people with advanced HIV disease should be given priority for assessment and initiation [4]. It is calculated as the difference between the date of ART enrollment and the date of HIV diagnosis.

### Health-seeking behavior

Any activity undertaken by individuals who perceive themselves to have a health problem or to be ill for the purpose of finding an appropriate remedy [24]. In this study, health-seeking behavior was considered appropriate if the individual had a medical checkup in the past year without any complaints, or if they visited a healthcare center (public or private) when experiencing a health issue [25–27]. Otherwise, the health-seeking behavior was considered inappropriate.

### Data collection tools and procedures

The data collection tool, consisting of a questionnaire administered through structured interviews and a checklist for abstracting data from clinical records, was created by examining various pieces of literature. The data were collected via an exit face-to-face interview and clinical record review. Five clinical nurses employed in ART clinics, along with two public health officers with prior experience, were designated as data enumerators and supervisors. The data enumerator’s Android device had the KoboCollect version 2.4 application installed, and the blank form was downloaded from the KoboToolbox server to collect data by filling out the blank forms.

### Data quality management

A language expert performed the translation of the questionnaire into the local language. The data enumerators and supervisors received thorough training on the study’s objectives and data collection methods with ample time for them to practice using KoboCollect. The data quality was ensured by examining completed questionnaires for critical items before uploading them to KoboToolbox. The principal investigator regularly verified the submitted files to ensure consistency and completeness. Finally, data completeness reassurance and data cleaning were conducted.

### Data processing and analysis

The data from the Enkoto web server were downloaded as an Excel file and imported into R version 4.3.3 (R Core Team, Vienna, Austria) for analysis [28]. Descriptive statistics were performed to describe the characteristics of the study participants. The backward elimination variable selection method was employed. The Akaike Information Criterion (AIC) was used to evaluate and select the best-fitting model to describe the relationship between AHD and the predictors. The selected model carries 98% of the cumulative model weight. In multivariable logistic regression, variables with a p-value <0.05 were deemed independent predictors. Multicollinearity was assessed using collinearity diagnostic statistics, including variance inflation factor (VIF) and tolerance tests. The Hosmer-Lemeshow test was used to check for model goodness-of-fit (p-value = 0.87). Finally, the data were presented in the form of tables and text.

### Ethics statement

All procedures described were performed in compliance with The Code of Ethics of the Declaration of Helsinki for experiments involving humans and have been approved by the Institutional Review Board of the College of Medical and Health Sciences, Dilla University, with project number duirb/035/23-05 on 18^th^ May 2023. Written informed consent was obtained from the study participants. The data were collected anonymously, kept confidential, and secured throughout the study.

## Results

### Baseline characteristics of the study participants

#### Sociodemographic characteristics

The mean (±SD) age of the study participants was 31.3 (±8.7) years [ranging from 18 to 62], with half (49.3%) falling within the age group of 30 to 49 years. More than half of the respondents were female (55.5%), lived in urban residences (51.4%), were married (59.5%), and had a primary education (55.0%). Approximately two-thirds of them had two or more children. The median (IQR) average monthly income was 1000 (400 to 3000) Ethiopian Birr (Table 1).

**Table 1:** Sociodemographic characteristics of newly diagnosed PLHIV-initiating ART in the Gedeo zone, southern Ethiopia, from May 29, 2023, to February 06, 2024 (n = 422).

Variables	Frequency (n)	Percent (%)
Category		
Age of respondents (in years)		
18–29	197	46.7
30–49	208	49.3
≥50	17	4.0
Sex		
Female	234	55.5
Male	188	44.5
Place of residence		
Rural	205	48.6
Urban	217	51.4
Ethnicity		
Gedeo	212	50.2
Oromo	114	27.0
Amhara	23	5.45
Sidama	32	7.6
Guraghe	18	4.3
Others^#^	23	5.45
Religion		
Protestant	230	54.5
Orthodox	144	34.1
Muslim	33	7.8
Catholic	1	0.3
Others^@^	14	3.3
Educational status		
No formal education	124	29.4
Primary education	232	55.0
Secondary and above education	66	15.6
Employment status		
Housewife	107	25.4
Private work	146	34.6
Public servant	27	6.4
Unemployed	47	11.1
Others^®^	95	22.5
Marital status		
Single	68	16.1
Married	251	59.5
Divorced	51	12.1
Widowed	52	12.3
Average monthly income in ETB (in USD)		
<400 (<4)	103	24.4
400–3000 (4–30)	229	54.3
>3000 (>30)	90	21.3
Number of children		
<2	156	37.0
≥2	266	63.0

Others^#^: Kore and Burji; Others^@^: traditional religions; Others^®^: daily laborer

#### Clinical and health system-related characteristics

The mean (±SD) BMI of the study participants was 19.6 (±2.5), with nearly two-thirds (65.6%) falling within the normal range. More than half (52.1%) had no opportunistic infections, and more than two-thirds (68.5%) had no comorbidities. Nearly two-thirds (65.4%) were diagnosed in the hospital, and a facility-based test modality was employed for the majority (82.2%) of participants. Rapid initiation of ART was offered for the majority (61.1%). Approximately two-thirds (63%) were noninsured by community-based health insurance (CHI). Most respondents (79.6%) indicated a preference for public healthcare providers over private healthcare providers (Table 2).

**Table 2:** Clinical and health system-related characteristics of newly diagnosed PLHIV-initiating ART in the Gedeo zone, southern Ethiopia, from May 29, 2023, to February 06, 2024 (n = 422).

Variables	Frequency (n)	Percent (%)
Category		
WHO clinical staging		
Stage 1	224	53.1
Stage 2	63	14.9
Stage 3	122	28.9
Stage 4	13	3.1
Body mass index (BMI)		
Underweight (BMI <18.5)	130	30.8
Normal (18.5–24.9)	277	65.6
Overweight/obese (BMI ≥25)	15	3.6
Functionality		
Working	345	81.8
Ambulatory	77	18.2
Opportunistic infections (OIs)		
Absent	220	52.1
Present	202	47.9
Comorbidity		
Absent	289	68.5
Present	133	31.5
Community-based health insurance		
Noninsured	266	63.0
Insured	156	37.0
Diagnosis cites		
Health center	129	30.6
Hospital	276	65.4
Project campaign	17	4.0
HIV-test modality		
Community-based (ICT)	75	17.8
Facility-based	347	82.2
Medical check-ups in the past year		
Yes	81	19.2
No	341	80.8
Visit healthcare centers for a health condition		
Yes	393	93.1
No	29	6.9
Preferred type of healthcare provider		
Private	86	20.4
Public	336	79.6

#### Psychosocial and behavioral characteristics

The majority (93.4%) exhibited appropriate health-seeking behavior. Approximately two-thirds (61.4%) of the participants reported not consuming alcohol, and similarly, the majority (92.2%) were nonsmokers. Approximately two-thirds (64.0%) had a history of facility-based HIV testing. Over two-thirds sought care from traditional or religious healers. A total of 60.4% had high social cohesion, and the proportion of social participation was 87% (Table 3).

**Table 3:** Psychosocial and behavioral characteristics of newly diagnosed PLHIV-initiating ART in the Gedeo zone, southern Ethiopia, from May 29, 2023, to February 06, 2024 (n = 422).

Variables	Frequency (n)	Percent (%)
Category		
Social trust		
Yes	259	61.4
No	163	38.6
Collective engagement		
Yes	287	68.0
No	135	32.0
Social participation		
Yes	367	87.0
No	55	13.0
Social cohesion		
High	255	60.4
Low	167	39.6
Health seeking behavior		
Appropriate	394	93.4
Inappropriate	28	6.6
Sought care from traditional/religious healer		
Yes	293	69.4
No	129	30.6
Smoking		
Smoker	33	7.8
Nonsmoker	389	92.2
Alcohol consumption		
Yes	163	38.6
No	259	61.4
Ever considered HIV testing		
Yes	195	46.2
No	227	53.8
History of facility-based HIV testing		
Yes	152	36.0
No	270	64.0

### Prevalence of advanced HIV disease

The study revealed that the overall prevalence of advanced HIV disease (AHD) among newly diagnosed individuals living with HIV who were initiating antiretroviral therapy (ART) in the Gedeo zone was 145 out of 422, corresponding to 34.4% (95% CI: 29.8%, 39.1%).

### Predictors of advanced HIV disease

In bivariable logistic regression analyses, twelve variables—namely, age, sex, place of residence, educational status, employment status, marital status, average monthly income, number of children, test modality used, social trust, social participation, and alcohol consumption—showed an association with a p-value <0.25 and were considered candidates for the multivariable logistic regression model. Health-seeking behavior is likely to lie on the causal pathway between many predictor variables and AHD, so it was excluded from the final model.

In multivariable logistic regression analysis, place of residence, alcohol consumption, and HIV-test modality used were identified as independent predictors of advanced HIV disease among newly diagnosed people living with HIV-initiating ART in the Gedeo zone. Accordingly, individuals from rural residential areas were 3.5 times more likely to present with advanced HIV disease (AHD) than were those from urban residential areas (AOR = 3.48, 95% CI: 2.24, 5.47). The likelihood of advanced HIV disease was 2.5 times greater among alcohol consumers than among nonconsumers (AOR = 2.48, 95% CI: 1.59, 3.90). Furthermore, PLHIV identified through community-based index case testing were 75% less likely to present with advanced HIV disease (AHD) than those identified through facility-based testing modalities (AOR = 0.26, 95% CI: 0.13, 0.51) (Table 4).

**Table 4:** Bivariable and multivariable logistic regression of predictors of AHD among newly diagnosed PLHIV-initiating ART in the Gedeo zone, southern Ethiopia from May 29, 2023, to February 06, 2024 (n = 422).

Variables	AHD		COR (95% CI)	P-value	AOR (95% CI)	P-value
Category	Yes	No				
Age (in years)						
18–29	56 (71.6)	141 (28.4)	1		1	
30–49	81 (61.1)	127 (38.9)	1.61 (1.06, 2.44)	0.025	1.42 (0.88, 2.29)	0.39
50 and above	8 (52.9)	9 (47.1)	2.24 (0.80, 6.14)		1.62 (0.53, 4.88)	
Sex						
Female	73 (31.2)	161 (68.8)	1		1	
Male	72 (38.3)	116 (61.7)	1.37 (0.91, 2.05)	0.127	1.34 (0.85, 2.12)	0.21
Place of residence						
Rural	96 (46.8)	109 (53.2)	3.02 (1.99, 4.62)	<0.001	**3.48 (2.24, 5.47)**	<0.001*
Urban	49 (22.6)	168 (77.4)	1		1	
Educational status						
No formal education	53 (42.7)	71 (57.3)	1			
Primary education (1–8)	71 (30.6)	161 (69.4)	0.59 (0.38, 0.93)	0.023	0.78 (0.43, 1.39)	0.92
Sec. and above education	21 (31.8)	45 (68.2)	0.63 (0.33, 1.16)		0.96 (0.42, 2.20)	
Employment status						
Housewife	42 (39.3)	65 (60.7)	1		1	
Private work	40 (27.4)	106 (72.6)	0.58 (0.34, 0.99)	0.048	0.54 (0.24, 1.24)	0.91
Public servant	7 (25.9)	20 (74.1)	0.54 (0.20, 1.34)		0.44 (0.11, 1.64)	
Unemployed	19 (40.4)	28 (59.6)	1.05 (0.52, 2.11)		1.05 (0.42, 2.63)	
Others^®^	37 (38.9)	58 (61.1)	0.99 (0.56, 1.74)		0.61 (0.26, 1.41)	
Marital status						
Single	17 (25.0)	51 (75.0)	1		1	
Divorced	22 (43.1)	29 (56.9)	2.28 (1.05, 5.03)	<0.001	1.77 (0.69, 4.59)	0.72
Married	77 (30.7)	174 (69.3)	1.33 (0.73, 2.50)		0.86 (0.37, 1.99)	
Widowed	29 (55.8)	23 (44.2)	3.78 (1.76, 8.36)		1.99 (0.74, 545)	
Average monthly income in ETB (in USD)						
<400 (<4)	45 (43.7)	58 (56.3)	1.63 (1.00, 2.62)	0.046	1.81 (1.04, 3.18)	0.44
>3000 (>30)	26 (28.9)	64 (71.1)	0.85 (0.49, 1.44)		1.28 (0.69, 2.38)	
400–3000 (4–30)	74 (32.3)	155 (67.7)	1		1	
Number of children						
<2	46 (29.5)	110 (70.5)	1		1	
≥2	99 (37.2)	167 (62.8)	1.42 (0.93, 2.18)	0.107	1.42 (0.87, 2.31)	0.16
HIV-test modality						
Community-based (ICT)	12 (16.0)	63 (84.0)	0.31 (0.15, 0.57)	<0.001	**0.26 (0.13, 0.51)**	<0.001*
Facility-based	133 (38.3)	214 (61.7)	1		1	
Social trust						
No	69 (42.3)	94 (57.7)	1		1	
Yes	76 (29.3)	183 (70.7)	0.57 (0.37, 0.85)	0.006	0.72 (0.46, 1.13)	0.15
Social participation						
No	13 (23.6)	42 (76.4)	1		1	
Yes	132 (36.0)	235 (64.0)	1.81 (0.96, 3.63)	0.076	1.86 (0.92, 3.97)	0.09
Alcohol consumption						
Yes	71 (43.6)	92 (56.4)	1.93 (1.28, 2.91)	0.001	**2.48 (1.59, 3.90)**	<0.001*
No	74 (28.6)	185 (71.4)	1		1	

Others^®^: daily laborer; ICT: index case testing; 1: reference category; CI: confidence interval; COR: crude odds ratio; AOR: adjusted odds ratio; *statistically significant at p-value <0.05

## Discussion

This study aimed to assess the prevalence and predictors of advanced HIV disease among newly diagnosed people living with HIV-initiating antiretroviral therapy in the Gedeo zone, southern Ethiopia. The prevalence of AHD was 34.4% (95% CI: 29.8%, 39.1%). Rural residence, alcohol consumption, and being identified through community-based index case testing (ICT) modality were identified as independent predictors of advanced HIV disease among newly diagnosed people living with HIV.

This study is the first to assess the predictors of advanced HIV disease among newly diagnosed PLHIV in Ethiopia. The prevalence of AHD in this study was consistent with studies conducted in South Africa (32.9%) [29], Kinshasa, the Democratic Republic of the Congo (32.0%) [30], Kampala, Uganda (35.1%) [20], Sierra Leone (41.6%) [21], and a tertiary-care hospital in Uganda (39.0%) [31]. On the other hand, the prevalence in the current study was lower than that in studies conducted in rural Ethiopia (60.4%) [32], Guinea-Bissau (48.7%) [33], semi-urban polyclinic in Epworth, Zimbabwe (47.4%) [34], the rural district of Mozambique (59.4%) [35], Senegal, West Africa (71.1%) [16]. Disparities in societal and cultural factors, study periods, settings, and healthcare systems might explain this discrepancy. Although Mozambique exhibited a higher prevalence, it is important to highlight two concerns. First, the study focused on evaluating the burden of AHD among high-risk PLHIV, specifically those with a TB index and TB contacts. Another noteworthy concern that could have contributed to the observed difference is that only 155 out of 673 HIV-positive individuals who consented were assessed for AHD.

The likelihood of presenting with AHD during antiretroviral initiation was 3.5 times greater for individuals from rural residences than for those from urban residences. Many underlying challenges in rural areas could contribute to this, including lower access to health care, late presentation to care, lower uptake of HIV care, and insufficient emotional support networks to overcome stigma. A study conducted in the West Arsi zone of Ethiopia reported that rural residents present late for HIV/AIDS care [36]. This finding is further supported by evidence from the developed world, where rural residence is associated with delayed entry into care [37]. In Ethiopia, it has been reported that rural residents have low utilization of HIV testing, which makes it more likely for them to seek HIV care at an advanced stage [38–40]. Similarly, the stigma surrounding HIV presents a significant obstacle to both men and women seeking testing and subsequently enrolling in HIV care [41, 42]. Our study highlights the importance of improving HIV screening initiatives in rural areas, as this can facilitate earlier detection of HIV infections among individuals. Early diagnosis enables prompt initiation of antiretroviral therapy (ART), which is crucial for effectively managing HIV infection and has the potential to enhance health outcomes. Community-based index case testing might help screen individuals in rural residences. Improving community sensitization efforts could lead to earlier antiretroviral therapy (ART) initiation, thus improving health outcomes [43]. Increasing HIV testing rates in rural communities through community health campaigns (CHCs) is a successful strategy for identifying undiagnosed HIV [44]. Researchers should investigate the social determinants underlying the disparity in AHD burden between rural and urban residents.

The likelihood of presenting with advanced HIV disease was 2.5 times greater among alcohol consumers than among non-consumers. In Ethiopia, alcohol consumption is strongly associated with late presentation to HIV care [36, 45, 46]. Presenting to care at an advanced stage is more common among late presenters than among early initiators. From an immunological perspective, drinking alcohol is associated with a decreased frequency of lymphocytes and an increased risk of both viral and bacterial infections [47]. This assertion is corroborated by a study indicating that continual unhealthy alcohol consumption is linked to more advanced HIV disease severity among HIV-infected individuals [48]. As a result of alcohol abuse, multiple immune components are suppressed, increasing the likelihood of infection. The pathology observed in individuals with HIV/AIDS can be directly attributed to immune suppression mechanisms caused by alcohol use [49]. Another possible explanation is that alcohol promotes the development of proinflammatory CD4+ T cells and decreases the differentiation of immunosuppressive CD4+ T cells [50]. Alcohol promotes pro-inflammatory immune responses as well as disrupts anti-inflammatory cytokine production. Furthermore, chronic alcohol consumption disrupts the optimal functioning of all components of the adaptive immune response, affecting both cell-mediated and humoral immune responses [51]. For alcohol consumers to have a favorable prognosis, they must receive comprehensive counseling about the detrimental effect of alcohol consumption on their treatment progress. This counseling should be on par with the guidance for adhering to antiretroviral therapy. Program designers should consider incorporating the burden of alcohol consumption into national guidelines.

People diagnosed with HIV through community-based index case testing had a 75% lower likelihood of presenting with advanced HIV disease (AHD) than those identified through the facility-based testing modality. This finding aligns with research from Lebanon, indicating that individuals screened for testing were notably less likely to present with an advanced stage than those tested at medical facilities [52]. The community-based testing modality makes a significant contribution to the HIV continuum of care. First, it is important for the earlier identification of HIV-positive individuals [53–56], which is crucial in the HIV care cascade [57]. Additionally, the community-based testing modality identifies HIV-positive people at higher CD4 counts than facility-based testing [53, 58, 59]. Evidence also suggests that the proportion of case detection (test yield) is greater in community-based testing than in standard facility-based testing [60–63]. Community-based testing followed by earlier ART initiation may result in better outcomes [64, 65] and prevent most HIV-related mortality [66].

In the context of a generalized HIV epidemic, the World Health Organization advocates for community-based HIV testing services that include linkage to prevention, treatment, and care services [67]. Furthermore, in high-prevalence settings, the inclusion of household-based active case finding should be considered as part of integrated HIV-TB care [65]. In high-impact HIV hotspots in Ethiopia, index case testing is the most effective and efficient tool for identifying HIV cases among key populations [60]. Index case testing has the potential to assist nations in sub-Saharan Africa in reaching the United Nations 95-95-95 goals [68–71]. Therefore, it is worthwhile to scale up ICT implementation across all communities through collaborative efforts involving all stakeholders in HIV programs, aiming to achieve the first 95-95-95 target, which calls for 95% of all people living with HIV to know their HIV status. This should be translated with caution, as people diagnosed through community-based testing modalities initiate ART at a lower rate than those diagnosed through clinical testing [72], and linkage to care after community-based testing may be a challenge [73].

Our study included individuals from diverse backgrounds who were initiating antiretroviral therapy at different levels of health care; however, the study is not without limitations. Because of the cross-sectional nature of the study, a cause-and-effect relationship could not be established. Variables such as the number of sexual partners and condom utilization might be associated with AHD but were not investigated in the current study. Future research on AHD could consider examining these variables. Furthermore, the clinical features of PLHIV presenting with AHD were not well examined in the current study, and this is a prospect for future research.

## Conclusions

Our study revealed that the burden of advanced HIV disease among newly diagnosed PLHIV-initiating ART in the Gedeo zone was considerable. Rural residence, alcohol consumption, and being identified through community-based index case testing were found to be independent predictors of AHD. PLHIV who consume alcohol should receive detailed counseling on how it can negatively impact their progress with antiretroviral treatment. HIV testing should be enhanced in rural communities by strengthening community health campaigns (CHCs) and community-based testing in high-impact HIV hotspots. These will help identify undiagnosed PLHIV early when CD4 cell counts are typically higher.

## Supporting information

S1 Checklist STROBE (Strengthening The Reporting of OBservational Studies in Epidemiology) checklist. (PDF)

S1 Dataset (XLSX)

## List of abbreviations

AHD: Advanced HIV Disease
AIC: Akaike Information Criterion
ART: Antiretroviral Therapy
CD4: Clusters of Differentiation 4
CHC: Community Health Campaigns
CHI: Community-Based Health Insurance
HC: Health Center
ICT: Index Case Testing
PEPFAR: The U.S. President’s Emergency Plan for AIDS Relief
PH: Primary Hospital
PICT: Provider-initiated Counseling and Testing
PLHIV: People Living with HIV
TB: Tuberculosis
VCT: Voluntary Counseling and Testing
WHO: World Health Organization
